# Effect of apremilast on entheseal bone damage in psoriasis patients at risk of developing psoriatic arthritis - data from the prospective, interventional EPOS study

**DOI:** 10.1186/s13075-026-03873-z

**Published:** 2026-08-08

**Authors:** David Simon, Ioanna Minopoulou, Sara Bayat, Louis Schuster, Koray Tascilar, Frank Roemer, Melek Yalcin Mutlu, Jürgen Rech, Dagmar Werner, Gerhard Krönke, Filippo Fagni, Michael Sticherling, Georg Schett, Arnd Kleyer

**Affiliations:** 1 Department of Internal Medicine 3-Rheumatology and Immunology, Friedrich- Alexander-University Erlangen-Nürnberg (FAU) and Universitätsklinikum Erlangen, Erlangen, Germany; 2 Deutsches Zentrum Immuntherapie (DZI), Friedrich-Alexander-University Erlangen-Nürnberg (FAU) and Universitätsklinikum Erlangen, Erlangen, Germany; 3 Department of Rheumatology and Clinical Immunology, Charité - Universitätsmedizin Berlin, Berlin, Germany; 4 Fraunhofer Institute for Translational Medicine and Pharmacology, Allergology and Immunology, Berlin, Germany; 5 Deutsches Rheuma-Forschungszentrum Berlin, Berlin, Germany; 6 Department of Radiology, Boston University School of Medicine, Boston, Massachusetts USA; 7 Institute of Radiology, Friedrich-Alexander-University Erlangen-Nürnberg (FAU) and Universitätsklinikum Erlangen, Erlangen, Germany; 8 Department of Dermatology, Friedrich-Alexander-University (FAU) Erlangen- Nürnberg and Universitätsklinikum Erlangen, Erlangen, Germany

## Abstract

**Background/Purpose:**

A subset of psoriasis (PsO) patients exhibits subclinical entheseal inflammation and bone remodeling placing them at higher risk of developing psoriatic arthritis (PsA). Whether early pharmacological interventions during this phase can modulate these inflammatory and structural changes remains largely unclear.

**Methods:**

In this single-arm, open-label trial (EPos, EUDRACT 2018-000335-27) PsO patients with moderate-to-severe psoriasis, arthralgia, subclinical inflammatory and/or structural bone changes assessed by hand MRI and/or HR-pQCT were included. Patients who had current or past signs of PsA or prior b/tsDMARD exposure were excluded. Participants received apremilast 30 mg BID over 24 weeks. The primary endpoint was the change in structural entheseal lesions (SEL) (number, density and structure) at hand joints assessed by HR-pQCT (week 24). Secondary endpoints were change in bone and inflammatory alterations assessed by MRI (PsAMRIS) as well as clinical response.Safety was monitored

**Results:**

Twenty patients (50.0±11.6 years;9 women) were included, all with long-standing PsO and frequent nail and scalp involvement. Over 24 weeks no significant progression in the SEL number was detected while entheseal cortical density and cortical thickness also remained stable. No progression in number and volume of erosions was observed. Total PsAMRIS as marker of inflammation remained stable. Significant improvements in skin disease activity (PASI: 10.9±6.7 vs. 5.2±6.6,p=0.013) and tender joint count (3.2±3.4 vs. 0.8±1.8,p=0.006) was seen. No new safety signals emerged.

**Conclusion:**

In PsO patients at increased risk of PsA, no significant change in subclinical entheseal bone or inflammatory imaging was observed over 24 weeks of apremilast treatment, while skin disease activity and pain outcomes improved. These findings support further investigation of disease-interception strategies in early psoriatic disease.

**Supplementary Information:**

The online version contains supplementary material available at 10.1186/s13075-026-03873-z.

## Key Message

What is already known on this topic

Subclinical entheseal inflammation and bone changes in psoriasis are associated with an increased risk of development of PsA and provide an opportunity for early intervention. What this study addsUsing high-resolution imaging, this study shows that apremilast can stabilize early entheseal structural and inflammatory changes while improving clinical outcomes in PsO patients at high risk of developing PsA

How this study might affect research, practice or policy

These findings support early disease interception strategies in psoriatic disease and highlight entheseal-focused imaging as a sensitive tool for risk stratification and monitoring preventive interventions.

**Supplementary Information:**

The online version contains supplementary material available at 10.1186/s13075-026-03873-z.

## Introduction

Psoriatic arthritis (PsA) is a chronic inflammatory musculoskeletal disease characterized by the coexistence of synovial and entheseal inflammation in combination with skin disease [1]. Epidemiological data indicate that approximately 20–30% of patients with psoriasis will develop PsA over time, with disease transition typically occurring gradually over several years [1]. Increasing evidence supports the concept of a prodromal or transition phase preceding clinically overt PsA, during which inflammatory processes may spread from the skin to the musculoskeletal system, particularly to joints and entheses [2]. During this transitional stage, patients frequently report arthralgia in the absence of clinically apparent joint swelling, while subclinical inflammatory and structural changes may already be present at entheseal sites [2–6]. Advanced imaging studies indicate that early PsA-related alterations, such as subclinical synovitis, enthesitis, osteitis, and microstructural bone remodeling, may precede clinically overt PsA. These changes are underrecognized in routine practice due to limited sensitivity of conventional imaging and lack of knowledge for defined anatomical target areas to scan in routine practice [4, 5, 7, 8]. Sensitive imaging techniques such as high-resolution peripheral computed tomography (HR-pQCT) are therefore essential to detect early pathological changes and may inform improved use of standard imaging modalities for early disease interception.

The pathogenesis driving this early transition phase from psoriasis to PsA involves a complex interplay of genetic, environmental, and immunological factors, marked by the migration of inflammatory cells from the skin to the joints and entheses [9]. Key cytokines like TNF-α, IL-17, and IL-23 play pivotal roles in driving inflammatory processes that characterizes both psoriasis and PsA. Macrophages and activated T cells, particularly Th1, Th17 or Tc17 cells, are central to this process, perpetuating the inflammatory response as they migrate to the synovio-entheseal complex [10, 11]. This inflammatory cascade leads to tissue damage and the characteristic bony proliferation at the entheses (so called structural entheseal lesions) [3, 5]. Genetic predispositions, such as HLA-B27 and IL23R, alongside environmental triggers, further influence this transition [12].

Recent research has improved our understanding of the transition phase and short-term as well as medium- to long-term risk factors for the development of PsA [13]. For example, a higher severity of skin disease, the presence of arthralgia, subclinical inflammation and the presence of structural entheseal lesions, increases the likelihood of developing PsA [2–6, 14]. The identification of such detrimental factors facilitates targeted intervention with the aim of reducing the risk of transition to PsA. Registry and cohort data of psoriasis patients and recent intervention trials in patients with subclinical PsA suggest that early treatment with biologic and targeted synthetic disease-modifying antirheumatic drugs (b/tsDMARDs) could be an effective approach to inhibit transition from psoriasis to PsA [15–21].

Apremilast, a phosphodiesterase 4 (PDE4) inhibitor, is effective in reducing cutaneous and musculoskeletal inflammation in psoriatic disease [22, 23]. However, its potential to intercept early in subclinical musculoskeletal disease has not been systematically investigated.

Hence, the aim of this prospective study was to evaluate the effect of apremilast on early subclinical musculoskeletal disease in psoriasis patients at high risk of developing psoriatic arthritis (PsA). We thereby comprehensively tested the effects of apremilast on structural entheseal and inflammatory changes in psoriasis patients with early signs of musculoskeletal disease by using HR-pQCT and magnetic resonance imaging (MRI).

## Methods

### Trial design and patients

EPos (Early Psoriatic Arthritis on Treatment Strategy) is a prospective, interventional, open-label, single-center clinical trial (EUDRACT No. 2018-000335-27) designed to evaluate the effects of apremilast on structural entheseal lesions, bone erosions, bone density and microstructure, biomechanical bone strength as well as early inflammatory changes in patients with psoriasis at high risk of developing PsA. The study was approved by the local ethics commission and by the Federal Institute for Drugs and Medical Devices (Bundesinstitut für Arzneimittel und Medizinprodukte) and was conducted at the University Hospital of Erlangen in accordance with the Declaration of Helsinki. Written informed consent was obtained from all participants prior to study enrollment.

Eligible participants were adults aged 18 years or older with moderate to severe psoriasis of at least six weeks’ duration before inclusion with indication for systemic therapy due to severity of skin involvement, who reported arthralgia and exhibited structural or inflammatory changes detected by hand MRI or HR-pQCT. Arthralgia was defined as patient-reported musculoskeletal pain and/or tenderness in the absence of clinically manifest arthritis, dactylitis, or clinically evident inflammatory musculoskeletal disease on rheumatological examination. Patients fulfilling the Classification Criteria for Psoriatic Arthritis (CASPAR), presenting with clinically manifest arthritis, dactylitis, or having received prior treatment with biologic or targeted synthetic disease-modifying antirheumatic drugs were excluded [24]. All participants were required to have received prior systemic therapy for psoriasis, either a conventional disease-modifying antirheumatic drug such as methotrexate or fumarate. Concomitant use of nonsteroidal anti-inflammatory drugs was permitted during the study.

Eligible patients were treated with apremilast at a dose of 30 mg twice daily for 24 weeks.

The primary endpoint was the change of structural entheseal lesions (number, density and structure) at week 24 assessed by HR-pCT. Secondary endpoints were inflammatory changes in the MRI, changes in bone structure and biomechanics and clinical response. Imaging was performed according to a standardized dominant-hand protocol and was not designed as symptom-directed imaging of all painful joints.

### Clinical and laboratory assessments

Demographic data such as age, sex, disease-specific parameters including skin disease duration, concomitant medication, and previous anti-inflammatory treatment (e.g. topical therapy, fumarate, corticosteroids, DMARD use), were recorded at baseline (BL). After screening, participants were evaluated at BL, week 12 and week 24. Clinical examination was performed for skin disease (Psoriasis Area and Severity Index (PASI), Body Surface Area (BSA)), tender entheseal count (LEI/MASES/SPARCC [25–27]), tender and swollen joint count (66/68). Patients’ and physicians’ global disease activity ratings (visual analogue scale, VAS) and patients’ pain intensity ratings (VAS) and patient-reported outcomes were collected (Health Assessment Questionnaire (HAQ), Psoriatic Arthritis Impact of Disease (PSAID)). In addition, blood samples were collected and analyzed to measure C-reactive protein (CRP), erythrocyte sedimentation rate (ESR), autoantibody status (anti-citrullinated protein antibodies (ACPA)). Adverse events (AEs) and serious AEs were documented.

### Imaging

Imaging was performed at the dominant hand as a standardized and reproducible peripheral site, because finger joints are commonly involved in early PsA and allow sensitive assessment of periarticular, entheseal, inflammatory, and structural bone changes by both HR-pQCT and MRI. This approach is supported by previous work demonstrating that structural entheseal lesions at the finger joints represent a characteristic feature of PsA-related bone pathology [3, 5].

### High-resolution peripheral quantitative computed tomography

HR-pQCT of the dominant hand was performed at inclusion and week 24 by a Xtreme CT 1 scanner (SCANCO Medical AG, Bruettisellen, Switzerland) following the manufacturer’s standard in vivo imaging protocol. The second and third metacarpophalangeal (MCP) joints and the distal radius of the same extremity were measured. MCP joint imaging was conducted as previously described, using 322 slices, with segmentation of the entire second metacarpal (MCP2) head and in a second step segmentation of the respective entheseal region [3, 28]. The distal radius was imaged using the manufacturer’s standard in vivo protocol, with acquisition initiated 9.5 mm proximal to a manually defined reference line and comprising 110 slices.

Prior to structural analysis and micro-finite element analysis (µFEA), all images were evaluated for motion artifacts, and only scans with motion grades 1 to 3 were included. Segmented regions encompassing the distal radius and the entheseal bone region at the MCP joints, as well as all other bone mineral density parameters of the MCP joints and radial bone, were registered using the manufacturer’s HR-pQCT longitudinal registration algorithm to ensure high-quality follow-up analyses.

**Fig. 1:**
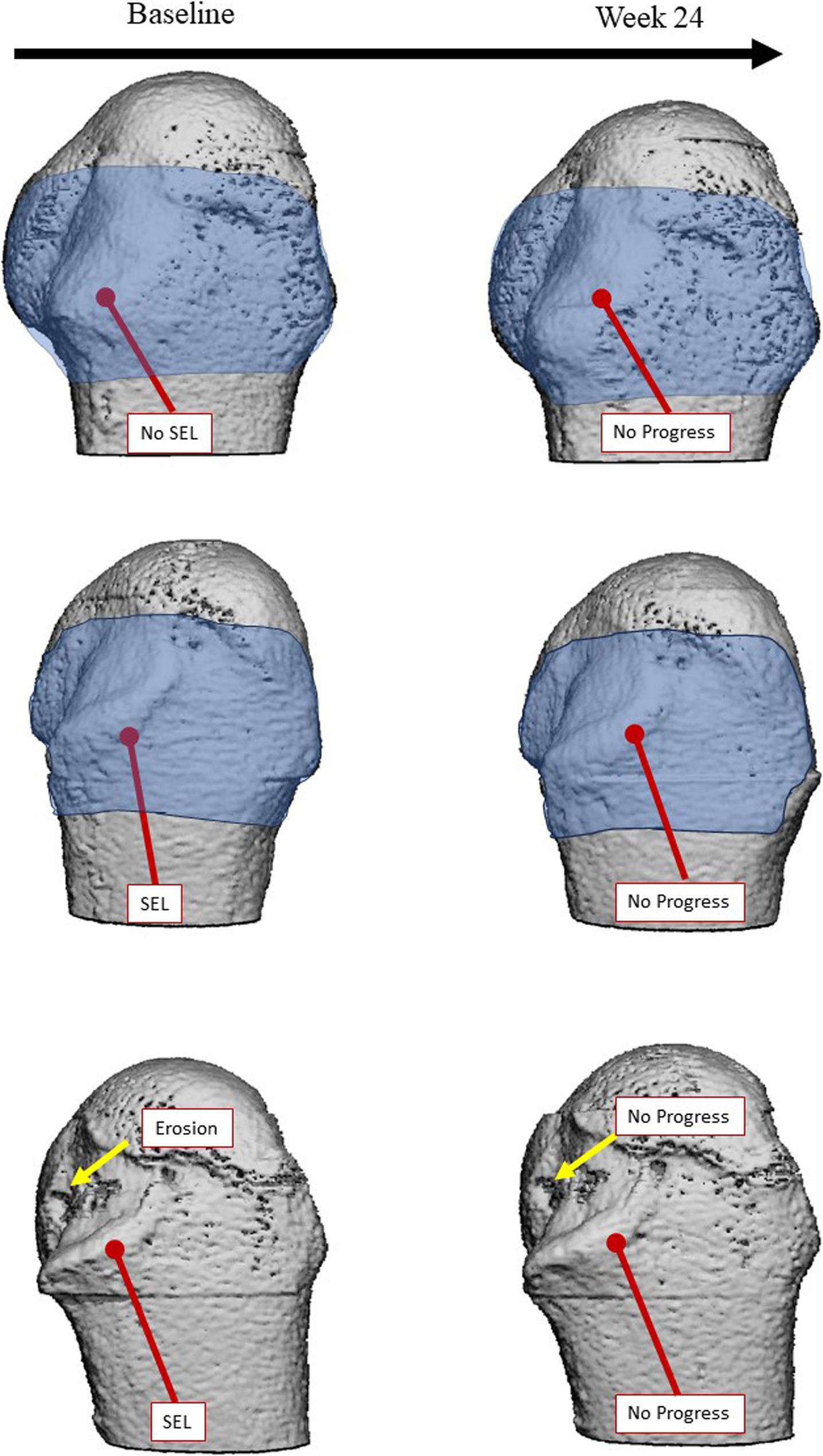
Visualization of structural entheseal lesions. Segmented dorsal-view images of MCP joints at baseline (left) and at the end of the study (week 24, right). Red arrows indicate structural entheseal lesions (SEL) and yellow arrows erosions. Row 1 shows no structural entheseal lesions at baseline and no progression at week 24, while row 2 demonstrates an entheseal lesion present at baseline that remains unchanged at week 24. Row 3 depicts a stable structural entheseal lesions (red arrow) and erosion (yellow arrow). Light blue transparent boxes indicate the defined entheseal region and MCP volume used for quantitative bone density and microstructural analyses

The primary endpoint was the change of structural entheseal lesions (number, density and structure). This endpoint was selected since this anatomically defined region represents a hallmark of entheseal bone pathology that differentiates PsA from other diseases [29]. Secondary endpoints were the quantitative characteristics of entheseal bone, including volumetric bone mineral density (vBMD) and microstructural parameters at the peripheral entheses of the second and third metacarpal heads. The corresponding method used to measure these entheseal bone parameters has been described in detail elsewhere [3, 30]. Briefly, entheseal lesions were evaluated using a combined qualitative and quantitative approach. First, after three-dimensional segmentation of the respective metacarpal bones, the number of structural entheseal lesions was visually identified and recorded by two independent readers (AK, LS) (Fig. 1). Readers were blinded to clinical data and to the chronological sequence of the imaging studies. Second, a quantitative assessment of entheseal bone was performed by measuring vBMD (mg HA/cm³) and microstructural parameters, including cortical thickness (mm), at both the entire metacarpal head and a predefined cortical-entheseal region. This combined approach enabled precise longitudinal quantification of the osteoproliferative burden and detailed characterization of their structural and density-related properties. By capturing microstructural entheseal bone changes that are not adequately detectable using conventional radiography, this methodology provides a robust and standardized framework for assesssing entheseal pathology.

Other secondary endpoints were number and volume of bone erosions (at the 2nd and 3rd metacarpal head). Number of erosions was recorded manually, and erosion volume (mm³) was quantified for the largest erosion (target erosion) using manual contouring and segmentation (LS). Additional secondary endpoints were vBMD and microstructure parameters of the intra-articular region of the MCP joints and distal radius: total vBMD); trabecular vBMD (Tb vBMD); cortical vBMD (all in mg/HA cm^3^ ); trabecular number/mm (Tb.N); trabecular thickness (Tb.TH) trabecular separation (Tb.Sp) and cortical thickness (mm).

In addition, bone biomechanical properties (distal radius; stiffness (kN/mm) and failure load (N)) were calculated using µFEA according to the manufacturer’s standard protocol, as previously described [3, 31].

### Magnetic resonance imaging

MRI of the dominant hand was performed at BL, and week 24, using a 1.5 Tesla scanner (Siemens Healthineers Magnetom Aera). Following a standardized scanning protocol, T1-weighted turbo spin echo (TSE) coronal and T1-weighted TSE fat-saturated transverse and coronal sequences after gadolinium injection, as well as T2-weighted turbo inversion recovery magnitude coronal and T2-weighted TSE fat-saturated coronal sequences, were used to assess synovitis, tenosynovitis, periarticular inflammation, bone marrow edema, bone proliferation and bone erosions. Scans were evaluated by two independent and blinded readers (SB, MYM) according to the OMERACT Psoriatic Arthritis Magnetic Resonance Imaging Score (PsAMRIS) [32]. Readers were blinded to clinical data and to the chronological sequence of the imaging studies. The total PsAMRIS score represents the sum of individual MRI features, including synovitis, tenosynovitis, periarticular inflammation, bone marrow edema (osteitis), erosions, and bone proliferation, reflecting overall structural damage and inflammatory burden. The total inflammation score comprises synovitis, tenosynovitis, osteitis, and periarticular inflammation, with periarticular inflammation scored based on the presence and extent of extracapsular inflammatory signal changes adjacent to the joint [33].

### Statistical analysis

Patient characteristics, laboratory, clinical, and MRI scores, and HR-pQCT results were reported as mean and SD or medians and quartile limits for continuous and as numbers and percentages for categorical variables. We also repeated analyses adjusting for BL age and sex with no material change in the findings and therefore omitted those analyses. Two-sided P values < 0.05 or two-sided 95% CIs of regression coefficients excluding 0 were considered significant. We did not use any specific procedures against multiple testing, and missing data were assumed to be missing at random. Study sample size was based on detecting a standardized mean difference of 0.6 between BL and follow-up for radial total vBMD, a difference that we have previously observed in Psoriatic Arthritis (PsA patients treated with bDMARDs compared with those treated with methotrexate [34]. A total of 24 patients would provide 80% power with a two-sided type-one error rate of 5% for this effect size. After accounting for a loss to follow-up of 20%, we planned to enroll 30 patients in this study. All statistical analyses were performed using the R statistical software (V4.2.1, R Foundation for Statistical Computing, Vienna, Austria).

## Results

### Patients

Twenty-eight patients were screened and 20 patients (9 women, 45%) with a mean age of 50.0 ± 11.6 years were included (6 screening failures, 2 withdrew informed consent before baseline). The mean duration of skin psoriasis was 18.6 ± 12.8 years. Participants had moderate-to-high psoriasis activity at baseline according to PASI (10.9 ± 6.7), with a mean BSA of 18.8 ± 12.9. Nail involvement was noted in 13 patients (65.0%) and a scalp involvement in 19 patients (95%). Mean VAS pain was 30.1 ± 23.3 mm. A mean of 3.2 ± 3.4 tender joints and 1.6 ± 2.7 tender entheses was noted. Per definition, no patient had joint swelling. Mean ± SD CRP levels were 4.6 ± 5.6 mg/L. Patient-reported outcomes indicated a mean HAQ score of 0.4 ± 0.4 and a mean PsAID score of 3.4 ± 1.8. No patient received concomitant conventional DMARD or systemic steroid treatment during the study period. A total of 80% percent of patients had previously been treated with methotrexate and or other csDMARD, and 20% percent had received dimethyl fumarate prior to the study. A summary of patient demographics and clinical characteristics is provided in (Table 1).

**Table 1:** Baseline characteristics

N	20
Age, mean (SD)	50.0 (11.6)
Sex	
Female, n (%)	9 (45.0)
Male, n (%)	11 (55.0)
BMI	30.37 (5.83)
Psoriasis disease duration, years mean (SD)	18.6 (12.8)
Smoking	
Formerly stopped, n (%)	7 (35.0)
Never, n (%)	10 (50.0)
Active smoker, n (%)	3 (15.0)
Psoriasis in family	
No, n (%)	13 (65.0)
Yes, n (%)	7 (35.0)
PsA in family	
No, n (%)	18 (90.0)
Yes, n (%)	2 (10.0)
Psoriasis	
Facial, n (%)	9 (45.0)
Scalp, n (%)	19 (95.0)
Genital, n (%)	9 (45.0)
Inverse, n (%)	9 (45.0)
Nail involvement, n (%)	13 (65.0)
Disease activity	
VAS pain, mm, mean SD	30.1 (23.3)
Swollen joints, n (mean SD)	0 (0)
Tender joints, n (mean SD)	3.2 (3.4)
Tender entheses, n (mean SD)	1.6 (2.7)
PASI, mean SD	10.9 (6.7)
BSA, mean SD	18.8 (12.9)
CRP, mg/L, (mean SD)	4.6 (5.6)
Patient reported outcomes	
HAQ, (mean SD)	0.4 (0.4)
PsAID, (mean SD)	3.4 (1.8)
Treatment	
csDMARDs	N 16 (80%)
Fumarates	N 4 (20%)
Phototherapy	N 5 (25%)
Topical treatment	N10 (50%)

*PASI*  Psoriasis Area and Severity Index, *HAQ*  Health Assessment Questionnaire, *PSAID*  Psoriatic Arthritis Impact of Disease, *csDMARDs*  conventional synthetic disease-modifying antirheumatic drug, *VAS*  Visual Analog Scale, *BSA*  Body Surface Area, *BMI* Body Mass Index

### Baseline imaging findings

HR-pQCT data for primary endpoint analysis (number of structural entheseal lesions) was available in 18 patients at BL and week 24. At BL, 4/18 (two HR-pQCT scans did not fulfil the imaging quality for analysis) patients showed structural entheseal lesions at the hand joints (Fig. 1). The mean total entheseal vBMD was 280.30 ± 64.60 mg HA/ cm³, cortical entheseal vBMD was 699.29 ± 92.66 mg HA/cm³ and the mean cortical entheseal thickness was 0.44 ± 0.15 mm (Table 2). 0.22 ± 0.94 bone erosions per patient were counted, with an average bone erosion volume of 0.88 ± 3.72 mm³ at MCP2. At the distal radius, mean total vBMD was 304.2 ± 73.13 mg HA/cm³, and mean cortical thickness was 0.71 ± 0.21 mm. Biomechanical assessment revealed a mean stiffness of 48,995.38 ± 16,360 kN/mm and a mean failure load of 2,331.82 ± 722.16 N.

**Table 2:** High-resolution peripheral quantitative computed tomography

Primary endpoint			
Structural entheseal lesions	Baseline	Week 24	*P* value
Available datasets (N)	18	18	
Structural entheseal lesions (N)	4/18	4/18	
Total entheseal vBMD (mg HA/cm^3^)	280.30 (64.60)	282.06 (66.97)	0.756
Cortical entheseal vBMD (mg HA/cm^3^)	699.29 (92.66)	701.81 (97.60)	0.523
Cortical entheseal thickness (mm)	0.44 (0.15)	0.44 (0.15)	0.323

**Secondary endpoints - metacarpophalangeal joints and radial bone**			
**Metacarpopahalgneal joint**	**Baseline**	**Week 24**	**P value**
Available datasets	18	17	
Erosions MCP 2			
Number (N)	0.22 (0.94)	0.24 (0.97)	n/a
Volume (mm^3^)	0.88 (3.72)	0.88 (3.64)	0,317
Erosions MCP 3			
Number (N)	0.22 (0.43)	0.41 (0.51)	0,083
Volume (mm^3^)	1.69 (3.90)	2.33 (3.72)	0,237
Available datasets	18	17	
Bone parameters MCP			
Cortical thickness	0.37 (0.15)	0.37 (0.15)	0,928
Total VBMD	290.06 (61.44)	292.35 (63.75)	0,469
Cortical VBMD	660.98 (101.24)	665.71 (101.41)	0,877

**Distal Radius**	**Baseline**	**Week 24**	**P value**
Available datasets	16	16	
Cortical thickness	0.71 (0.21)	0.71 (0.22)	0.905
Total vBMD	304.20 (73.13)	307.75 (77.32)	0.256
Cortical vBMD	809.77 (60.18)	817.74 (67.81)	0.501
Inner trabecular vBMD	138.32 (48.20)	138.55 (47.63)	0.679
Meta trabecular vBMD	233.70 (47.98)	234.14 (47.48)	0.955
Trabecular vBMD	177.19 (47.61)	177.60 (46.99)	0.959
Meta/inner trabecular vBMD ratio	2.00 (1.17)	1.96 (1.01)	0.45
Trabecular bone volume fraction	0.15 (0.04)	0.15 (0.04)	0,855
Trabecular network inhomogeneity	0.18 (0.09)	0.18 (0.09)	0.776
Trabecular number	2.13 (0.40)	2.12 (0.43)	0.816
Trabecular separation	0.42 (0.14)	0.42 (0.13)	0.776
Trabecular thickness	0.08 (0.03)	0.07 (0.01)	0.836
Cortical perimeter	77.84 (9.56)	77.66 (9.89)	0.864
Cortical thickness	0.71 (0.21)	0.71 (0.22)	0.905

**Biomechanics (distal radius)**	**Baseline**	**Week 24**	**P value**
Failure load	2331.82 (722.16)	2352.98 (664.25)	0.438
Stiffness	48995.38 (16360.48)	48921.31 (14532.92)	0.959

Values are given as means and standard deviations. vBMD = *volumetric bone mineral density*; N = *number* (or *Newton* when referring to biomechanical force parameters); kN = *kilonewton*; MCP = *metacarpophalangeal joint*

Baseline MRI assessments identified at least one inflammatory lesion in 9 of 19 evaluable scans. Synovitis was the most frequent finding, observed in 6 of 19 patients, followed by tenosynovitis in 4 of 19, osteitis in 2 of 19, and periarticular inflammation in 2 of 19 patients. The mean PsAMRIS for synovitis was 0.58 ± 1.12, for tenosynovitis 0.47 ± 1.17, for bone marrow edema 0.158 ± 0.69 and for periarticular inflammation 0.26 ± 0.93. The total PsAMRIS had a mean value of 2.74 ± 2.62.

### Effect of apremilast treatment on entheseal bone structure

By week 24, 18 h-pQCT data sets were available for numbers and 17 for quantitative measures of structural entheseal lesions. No significant changes in the number or characteristics of structural entheseal lesions were observed over the study period. The number of entheseal lesions remained stable (*N* = 4). Quantitative assessment of these lesions demonstrated no significant longitudinal changes in total entheseal vBMD, cortical entheseal vBMD, or cortical entheseal thickness. At week 24, total entheseal vBMD was 282.06 ± 66.97 mg/cm³ (*p* = 0.756), cortical entheseal vBMD was 701.81 ± 97.60 mg/cm³ (*p* = 0.523), and cortical entheseal thickness was 0.44 ± 0.15 mm (*p* = 0.323).

### Effect on bone erosions and composition of radial bone

Markers of osteodestruction remained unchanged during follow-up. The number of erosions (0.24 ± 0.97) and erosion volume (0.88 ± 3.64 mm³; *p* = 0.317) showed no significant change. At week 24, mean total vBMD was 307.75 ± 77.32 mg HA/cm³ (*p* = 0.256), and mean cortical thickness was 0.71 ± 0.22 mm (*p* = 0.905). In line with the absence of significant density or structural progression at the distal radius, biomechanical properties also remained stable. At week 24, mean failure load was 2,352.09 ± 664.25 N (*p* = 0.428), and mean stiffness was 48,921.31 ± 14,532.92 kN/mm (*p* = 0.959).

### MRI-detected inflammatory changes remained overall unchanged

MRI-based assessment of inflammatory lesions demonstrated no significant change of subclinical inflammation such as synovitis or tenosynovitis over the study period. Synovitis scores at week 24 were 0.71 ± 1.53 and did not differ significantly from baseline (*p* = 0.414). No tenosynovitis or periarticular inflammation was detected at follow-up, only osteitis increased with a mean score of 0.18 ± 0.73 at week 24 (*p* > 0.99). Consistent with these observations, the total PsAMRIS score showed no significant change over time (2.88 ± 2.98; *p* = 0.384). In contrast to the baseline assessment, no tenosynovitis or periarticular inflammation on MRI (0/17 patients) was observed at week 24. Number of patients with osteitis (*N* = 1) and synovitis (*N* = 5) remained stable. Compared with baseline, four additional patients showed MRI-detected erosions at follow-up (10/17). Detailed quantitative results for all assessed parameters are presented in (Table 3).

**Table 3:** Magnetic resonance imaging

Available datasets (*N*)	Baseline (mean SD)	Week 24 (mean SD)	*P* value
	19	17	
Osteitis	0.158 (0.688)	0.176 (0.728)	> 0.99
Peri-articular inflammation	0.263 (0.933)	0.00 (0.00)	0.317
Synovitis	0.579 (1.121)	0.706 (1.532	0.414
Tenosynovitis	0.474 (1.172)	0.000 (0.00)	0.180
Total PSAMRIS Score	2.737 (2.621)	2.882 (2.977)	0.384
Total Inflammation score	1.474 (2.590)	0.882 (1.728)	0.734

Magnetic Resonance Imaging findings were evaluated using the PsAMRIS (Psoriatic Arthritis Magnetic Resonance Imaging Score)

### Effects of apremilast treatment on psoriatic skin disease and musculoskeletal symptoms

No patient developed PsA during the study. The number of tender joints (TJC 68) dropped significantly to 0.9 ± 1.9 (*p* = 0.010), the number of tender entheses was reduced to 0.5 ± 0.9 (*p* = 0.083), indicating a trend towards improvement, though not statistically significant. Mean VAS pain decreased to 23.9 ± 27.9 (*p* = 0.569). Mean PASI as well as the BSA significantly improved (4.7 ± 6.6 (*p* = 0.015); 7.9 ± 9.5 (*p* = 0.027)), reflecting a reduction in psoriasis activity (Table 4).

**Table 4:** Clinical endpoints

Patient Reported Outcomes	Baseline (mean SD)	Week 24 (mean SD)	*P* value
Pain global (0-100)	30.1 (23.3)	23.9 (27.9)	0.569
Patient global (0-100)	44.8 (25.4)	39.2 (29.2)	0.554
HAQ	0.4 (0.4)	0.3 (0.4)	0.073
Physician global (0-100)	37.6 (18.7)	22.3 (23.4)	0.055
Clinical Joint Outcomes			
Tender joint count (68)	3.2 (3.4)	0.9 (1.9)	0.01
Swollen joint count (66)	0.0 (0.0)	0.1 (0.3)	0.157
DAPSA	14.0 (7.6)	10.4 (7.2)	0.193
Clinical Entheseal Outcomes			
LEI	0.6 (1.1)	0.4 (0.8)	0.75
MASES	0.3 (0.7)	0.1 (0.2)	0.194
SPARCC	1.4 (2.4)	0.5 (0.9)	0.156
Clinical Skin Outcomes			
PASI	10.9 (6.7)	4.7 (6.6)	0.015
BSA	18.8 (12.9)	7.9 (9.5)	0.027
PSAID	3.4 (1.8)	3.2 (2.5)	0.276
Laboratory Parameters			
C-reactive Protein	4.6 (5.6)	3.3 (2.7)	0.246
Erythrocyte Sedimentation Rate	12.4 (10.6)	12.5 (9.6)	0.166

*PSAID *Psoriatic Arthritis Impact of Disease,* BSA *Body Surface Area,* and PASI *Psoriasis Area and Severity Index,were used to assess disease impact and skin involvement; enthesitis was evaluated using* LEI *Leeds Enthesitis Index,* MASES *Maastricht Ankylosing Spondylitis Enthesitis Score,and the* SPARCC *Spondyloarthritis Research Consortium of Canada,* Enthesitis Index, *overall disease activity and function were assessed using *DAPSA *Disease Activity index for Psoriatic Arthritis* and HAQ *Health Assessment Questionnaire, physician global assessment, patient global assessment, and pain were recorded on a 0-100 visual analog scale* VAS, *systemic inflammation was assessed by C-

### Safety

During the trial 44 mild and 9 moderate adverse events occurred with gastrointestinal disorders (*N* = 24) and infections (*N* = 9) being the most prominent adverse events. No new safety signals were detected. Supplement Fig. 1 shows number of adverse and serious adverse events during the trial.

## Discussion

The EPos study represents the first prospective interventional trial investigating the effects of apremilast on structural entheseal changes in a psoriasis population at increased risk of developing PsA.

Several findings are of importance: First, inhibition of phosphodiesterase-4 in b/tsDMARD naïve high-risk psoriasis patients is safe and effective and leads to improvement in both the skin and musculoskeletal domains as well as significant reduction in pain. These findings underline the reported clinical efficacy of apremilast in psoriasis and PsA. Second, these data show that no significant longitudinal change in HR-pQCT-derived structural entheseal lesions, entheseal bone density, cortical entheseal thickness, or HR-pQCT-assessed erosion number and volume was observed in this high-risk cohort of patients over a period of 24 weeks. However, MRI detected erosions in four additional patients at follow-up, indicating that structural imaging findings were not uniformly unchanged across modalities.

PsA is associated with a significant disease burden characterized by irreversible joint damage and reduced quality of life. As there is currently no cure for PsA and no medication that makes osteoproliferation disappear, early diagnosis and treatment is of utmost importance to prevent functional joint impairment [35, 36]. However, treatment of PsA is often started lateleaving patients exposed to a harmful inflammatory environment for several years, resulting in low to moderate remission rates [2, 37]. Selecting the right patients for early intervention can be challenging as the rate of progression to PsA in an unselected psoriasis population is generally low, ranging from 0.3 to 3 events per 100 patient-years [13]. Furthermore, due to the paucity of evidence, it is difficult to assess which treatment approach can successfully hinder PsA development.

Previous data on prospective, interventional trials in psoriasis patients at high risk are limited, the only completed interception trial using imaging as a primary endpoint biomarker, IVEPSA demonstrated the effect of IL-17 inhibition by secukinumab on bone and inflammation [20]. However, IVEPSA did not focus on structural entheseal lesions in particular. Nevertheless, the results of the EPos study were comparable to those of the IVEPSA trial, in which similar inclusion and exclusion criteria were applied. This raises the hypothesis that targeted b/tsDMARD therapy may be effective in stopping the disease early and halting the progression of subclinical changes. In addition, the results of this second interception study consolidate the definition of high-risk cohorts and form the basis for the definition of inclusion and exclusion criteria for future prospective intervention studies.

EPos has several limitations that should be considered when interpreting the findings. First, the relatively small sample size and the single-arm, open-label design limit the generalizability of the results and preclude definitive causal inferences. The absence of a control group introduces the potential for expectation and observer bias, which cannot be fully excluded in an open-label setting. Moreover, the study was not designed to directly assess the prevention of PsA but rather to explore the biological effects of apremilast on subclinical inflammatory and structural changes. Consequently, no valid conclusions can be drawn regarding whether apremilast reduces the long-term likelihood of PsA onset. MRI findings should also be interpreted cautiously, as total PsAMRIS did not significantly change over 24 weeks and individual inflammatory features showed mixed descriptive changes rather than a consistent improvement. Another limitation is that imaging was performed using a standardized dominant-hand protocol rather than symptom-directed imaging of every painful joint or entheseal site. Therefore, inflammatory changes at symptomatic sites outside the imaged hand may have been missed. In addition, ultrasound was not performed, therefore, we cannot assess ultrasound-detectable inflammation, or its progression over time. However, the standardized imaging approach enabled reproducible longitudinal assessment of predefined entheseal, periarticular, inflammatory, and structural bone changes in an anatomical region commonly involved in early PsA.

A strength of this study is the targeted assessment of bone changes at predefined peripheral entheses using high-resolution imaging enabling sensitive and reliable longitudinal evaluation of early microstructural changes that are not captured by conventional imaging. By selecting a precise, anatomy-driven imaging endpoint, this study was specifically designed to capture and quantify subtle changes in entheseal bone alterations relevant in an early or inception-risk population [3, 5]. By emphasizing the entheseal bone compartments rather than purely intra-articular changes, this approach enabled sensitive detection of early structural pathology. Further strengths include the well-defined cohort of psoriasis patients at high risk for PsA, which minimizes biases commonly encountered in registry-based studies, such as coding inaccuracies, selection bias, and unmeasured confounding. The controlled, prospective design further enhances internal validity and aligns with a precision medicine approach aimed at early disease interception. Finally, the exploratory nature of this trial provides an important foundation for future research. Larger, randomized controlled studies with longer follow-up are required to confirm these findings and to determine whether apremilast or other targeted interventions can effectively prevent or delay the onset of PsA in high-risk populations. Encouragingly, such trials have already been initiated and are expected to generate further evidence to refine early intervention strategies in psoriasis patients at risk of PsA [38].

In conclusion, the EPos trial underscores the potential that targeted interventions at the earliest phases of PsA not only improve psoriatic skin disease and early nociceptive manifestions but may be accompanied by no significant longitudinal change in selected HR-pQCT-derived structural changes associated with psoriatic disease, including structural entheseal lesions and measures of bone volume and bone quality. Larger randomized controlled studies with longer follow-up are required to determine whether early targeted intervention can modify imaging progression or reduce the risk of transition to PsA.

## Supplementary Information

Supplementary Material 1.

## Data Availability

All data relevant to the study are included in the article or uploaded as online supplemental information.

## References

[1] Ritchlin CT, Colbert RA, Gladman DD. Psoriatic Arthritis. N Engl J Med. 2017;376(10):957–70. 28273019 10.1056/NEJMra1505557

[2] Scher JU, Ogdie A, Merola JF, Ritchlin C. Preventing psoriatic arthritis: focusing on patients with psoriasis at increased risk of transition. Nat Rev Rheumatol. 2019;15(3):153–66. 30742092 10.1038/s41584-019-0175-0

[3] Simon D, Tascilar K, Kleyer A, Bayat S, Kampylafka E, Sokolova MV, et al. Association of Structural Entheseal Lesions With an Increased Risk of Progression From Psoriasis to Psoriatic Arthritis. Arthritis Rheumatol. 2022;74(2):253–62. 32103639 10.1002/art.41239

[4] Faustini F, Simon D, Oliveira I, Kleyer A, Haschka J, Englbrecht M, et al. Subclinical joint inflammation in patients with psoriasis without concomitant psoriatic arthritis: a cross-sectional and longitudinal analysis. Ann Rheum Dis. 2016;75(12):2068–74. 26916344 10.1136/annrheumdis-2015-208821

[5] Simon D, Faustini F, Kleyer A, Haschka J, Englbrecht M, Kraus S, et al. Analysis of periarticular bone changes in patients with cutaneous psoriasis without associated psoriatic arthritis. Ann Rheum Dis. 2016;75(4):660–6. 25653201 10.1136/annrheumdis-2014-206347

[6] Zabotti A, McGonagle DG, Giovannini I, Errichetti E, Zuliani F, Zanetti A, et al. Transition phase towards psoriatic arthritis: clinical and ultrasonographic characterisation of psoriatic arthralgia. RMD Open. 2019;5(2):e001067–e. 31749987 10.1136/rmdopen-2019-001067 PMC6827749

[7] Minopoulou I, Kleyer A, Yalcin-Mutlu M, Fagni F, Kemenes S, Schmidkonz C, et al. Imaging in inflammatory arthritis: progress towards precision medicine. Nat Rev Rheumatol. 2023;19(10):650–65. 37684361 10.1038/s41584-023-01016-1

[8] Zabotti A, McGonagle DG, Giovannini I, Errichetti E, Zuliani F, Zanetti A, et al. Transition phase towards psoriatic arthritis: clinical and ultrasonographic characterisation of psoriatic arthralgia. RMD Open. 2019;5(2):e001067. 31749987 10.1136/rmdopen-2019-001067 PMC6827749

[9] Raimondo MG, Mohammadian H, Angeli MR, Alivernini S, Fedorchenko V, Huang K, et al. Skin-derived myeloid precursors and joint-resident fibroblasts spread psoriatic disease from skin to joints. Nat Immunol. 2026;27(1):35–47. 41482544 10.1038/s41590-025-02351-z PMC12764428

[10] McGonagle D, Lories RJ, Tan AL, Benjamin M. The concept of a synovio-entheseal complex and its implications for understanding joint inflammation and damage in psoriatic arthritis and beyond. Arthritis Rheum. 2007;56(8):2482–91. 17665450 10.1002/art.22758

[11] Graßhoff H, Comdühr S, Nording H, von Esebeck J, Letz P, Prohaczka M, et al. Transition from psoriasis to psoriatic arthritis is characterized by distinct alterations in peripheral blood Tc17, Th17 and CD4 + TEM cells. Arthritis Rheumatol. 2025;78(2):332–43. 10.1002/art.43396 PMC12936891 40955716

[12] Schett G, Rahman P, Ritchlin C, McInnes IB, Elewaut D, Scher JU. Psoriatic arthritis from a mechanistic perspective. Nat Rev Rheumatol. 2022;18(6):311–25. 35513599 10.1038/s41584-022-00776-6

[13] Zabotti A, De Marco G, Gossec L, Baraliakos X, Aletaha D, Iagnocco A, et al. EULAR points to consider for the definition of clinical and imaging features suspicious for progression from psoriasis to psoriatic arthritis. Ann Rheum Dis. 2023;82(9):1162. 37295926 10.1136/ard-2023-224148

[14] Zabotti A, De Marco G, Gossec L, Baraliakos X, Aletaha D, Iagnocco A, et al. EULAR points to consider for the definition of clinical and imaging features suspicious for progression from psoriasis to psoriatic arthritis. Ann Rheum Dis. 2023;82(9):1162–70. 37295926 10.1136/ard-2023-224148

[15] Gisondi P, Bellinato F, Targher G, Idolazzi L, Girolomoni G. Biological disease-modifying antirheumatic drugs may mitigate the risk of psoriatic arthritis in patients with chronic plaque psoriasis. Ann Rheum Dis. 2022;81(1):68–73. 34144965 10.1136/annrheumdis-2021-219961

[16] Acosta Felquer ML, LoGiudice L, Galimberti ML, Rosa J, Mazzuoccolo L, Soriano ER. Treating the skin with biologics in patients with psoriasis decreases the incidence of psoriatic arthritis. Ann Rheum Dis. 2022;81(1):74–9. 34281904 10.1136/annrheumdis-2021-220865

[17] Singla S, Putman M, Liew J, Gordon K. Association between biological immunotherapy for psoriasis and time to incident inflammatory arthritis: a retrospective cohort study. Lancet Rheumatol. 2023;5(4):e200–7. 38251522 10.1016/S2665-9913(23)00034-6

[18] Solmaz D, Ehlebracht A, Karsh J, Bakirci S, McGonagle D, Aydin SZ. Evidence that systemic therapies for psoriasis may reduce psoriatic arthritis occurrence. Clin Exp Rheumatol. 2020;38(2):257–61. 31287403

[19] Rosenthal YS, Schwartz N, Sagy I, Pavlovsky L. Incidence of Psoriatic Arthritis Among Patients Receiving Biologic Treatments for Psoriasis: A Nested Case-Control Study. Arthritis & Rheumatology (Hoboken, NJ). 2022;74(2):237–43. 10.1002/art.41946 34423909

[20] Kampylafka E, Simon D, d’Oliveira I, Linz C, Lerchen V, Englbrecht M, et al. Disease interception with interleukin-17 inhibition in high-risk psoriasis patients with subclinical joint inflammation-data from the prospective IVEPSA study. Arthritis Res Ther. 2019;21(1):178. 31349876 10.1186/s13075-019-1957-0 PMC6659205

[21] Watad A, Zabotti A, Patt YS, Gendelman O, Dotan A, Ben-Shabat N, et al. From Psoriasis to Psoriatic Arthritis: Decoding the Impact of Treatment Modalities on the Prevention of Psoriatic Arthritis. Rheumatol Therapy. 2024;11(4):963–76. 10.1007/s40744-024-00680-3 PMC11264659 38847993

[22] Nash P, Ohson K, Walsh J, Delev N, Nguyen D, Teng L, et al. Early and sustained efficacy with apremilast monotherapy in biological-naïve patients with psoriatic arthritis: a phase IIIB, randomised controlled trial (ACTIVE). Ann Rheum Dis. 2018;77(5):690–8. 29343507 10.1136/annrheumdis-2017-211568 PMC5909747

[23] Kavanaugh A, Mease PJ, Gomez-Reino JJ, Adebajo AO, Wollenhaupt J, Gladman DD, et al. Treatment of psoriatic arthritis in a phase 3 randomised, placebo-controlled trial with apremilast, an oral phosphodiesterase 4 inhibitor. Ann Rheum Dis. 2014;73(6):1020–6. 24595547 10.1136/annrheumdis-2013-205056 PMC4033106

[24] Taylor W, Gladman D, Helliwell P, Marchesoni A, Mease P, Mielants H. Classification criteria for psoriatic arthritis: development of new criteria from a large international study. Arthritis Rheum. 2006;54(8):2665–73. 16871531 10.1002/art.21972

[25] Maksymowych WP, Mallon C, Morrow S, Shojania K, Olszynski WP, Wong RL, et al. Development and validation of the Spondyloarthritis Research Consortium of Canada (SPARCC) Enthesitis Index. Ann Rheum Dis. 2009;68(6):948–53. 18524792 10.1136/ard.2007.084244

[26] Healy PJ, Helliwell PS. Measuring clinical enthesitis in psoriatic arthritis: assessment of existing measures and development of an instrument specific to psoriatic arthritis. Arthritis Rheum. 2008;59(5):686–91. 18438903 10.1002/art.23568

[27] Heuft-Dorenbosch L, Spoorenberg A, van Tubergen A, Landewé R, van ver Tempel H, Mielants H, et al. Assessment of enthesitis in ankylosing spondylitis. Ann Rheum Dis. 2003;62(2):127–32. 12525381 10.1136/ard.62.2.127 PMC1754445

[28] Simon D, Kleyer A, Stemmler F, Simon C, Berlin A, Hueber AJ, et al. Age- and Sex-Dependent Changes of Intra-articular Cortical and Trabecular Bone Structure and the Effects of Rheumatoid Arthritis. J Bone Min Res. 2017;32(4):722–30. 10.1002/jbmr.3025 27787923

[29] Folle L, Simon D, Tascilar K, Krönke G, Liphardt AM, Maier A, et al. Deep Learning-Based Classification of Inflammatory Arthritis by Identification of Joint Shape Patterns-How Neural Networks Can Tell Us Where to Deep Dive Clinically. Front Med (Lausanne). 2022;9:850552. 35360728 10.3389/fmed.2022.850552 PMC8960274

[30] Simon D, Kleyer A, Englbrecht M, Stemmler F, Simon C, Berlin A, et al. A comparative analysis of articular bone in large cohort of patients with chronic inflammatory diseases of the joints, the gut and the skin. Bone. 2018;116:87–93. 30048820 10.1016/j.bone.2018.07.017

[31] Stemmler F, Simon D, Liphardt AM, Englbrecht M, Rech J, Hueber AJ, et al. Biomechanical properties of bone are impaired in patients with ACPA-positive rheumatoid arthritis and associated with the occurrence of fractures. Ann Rheum Dis. 2018;77(7):973–80. 29475856 10.1136/annrheumdis-2017-212404 PMC6029639

[32] Ostergaard M, McQueen F, Wiell C, Bird P, Bøyesen P, Ejbjerg B, et al. The OMERACT psoriatic arthritis magnetic resonance imaging scoring system (PsAMRIS): definitions of key pathologies, suggested MRI sequences, and preliminary scoring system for PsA Hands. J Rheumatol. 2009;36(8):1816–24. 19671819 10.3899/jrheum.090352

[33] ØStergaard M, McQueen F, Wiell C, Bird P, BØYesen P, Ejbjerg BO, et al. The OMERACT Psoriatic Arthritis Magnetic Resonance Imaging Scoring System (PsAMRIS): Definitions of Key Pathologies, Suggested MRI Sequences, and Preliminary Scoring System for PsA Hands. J Rhuematol. 2009;36(8):1816. 10.3899/jrheum.090352 19671819

[34] Simon D, Kleyer A, Bayat S, Tascilar K, Kampylafka E, Meinderink T, et al. Effect of disease-modifying anti-rheumatic drugs on bone structure and strength in psoriatic arthritis patients. Arthritis Res Ther. 2019;21(1):162. 31269973 10.1186/s13075-019-1938-3 PMC6607518

[35] Haroon M, Gallagher P, FitzGerald O. Diagnostic delay of more than 6 months contributes to poor radiographic and functional outcome in psoriatic arthritis. Ann Rheum Dis. 2015;74(6):1045–50. 24525911 10.1136/annrheumdis-2013-204858

[36] Snoeck Henkemans SVJ, de Jong PHP, Luime JJ, Kok MR, Tchetverikov I, Korswagen L-A, et al. Window of opportunity in psoriatic arthritis: the earlier the better? RMD Open. 2024;10(1):e004062. 38413172 10.1136/rmdopen-2023-004062 PMC10900390

[37] Smolen JS, Siebert S, Korotaeva TV, Selmi C, Bergmans P, Gremese E, et al. Effectiveness of IL-12/23 inhibition (ustekinumab) versus tumour necrosis factor inhibition in psoriatic arthritis: observational PsABio study results. Ann Rheum Dis. 2021;80(11):1419–28. 34162594 10.1136/annrheumdis-2021-220263 PMC8522461

[38] Haberman RH, MacFarlane KA, Catron S, Samuels J, Blank RB, Toprover M, et al. Efficacy of guselkumab, a selective IL-23 inhibitor, in Preventing Arthritis in a Multicentre Psoriasis At-Risk cohort (PAMPA): protocol of a randomised, double-blind, placebo controlled multicentre trial. BMJ Open. 2022;12(12):e063650. 36564123 10.1136/bmjopen-2022-063650 PMC9791418

